# Higher Ratios of Hyaluronic Acid Enhance Chondrogenic Differentiation of Human MSCs in a Hyaluronic Acid–Gelatin Composite Scaffold

**DOI:** 10.3390/ma9050381

**Published:** 2016-05-17

**Authors:** Christian G. Pfeifer, Arne Berner, Matthias Koch, Werner Krutsch, Richard Kujat, Peter Angele, Michael Nerlich, Johannes Zellner

**Affiliations:** 1 Department of Trauma Surgery, University Medical CentreRegensburg, Franz-Josef-Strauss-Allee 11, Regensburg 93053, Germany; arne.berner@ukr.de (A.B.); matthias.koch@ukr.de (M.K.); werner.krutsch@ukr.de (W.K.); richard.kujat@ukr.de (R.K.); peter.angele@sporthopaedicum.de (P.A.); michael.nerlich@ukr.de (M.N.); johannes.zellner@ukr.de (J.Z.); 2 Sporthopaedicum, Straubing/Regensburg, Hildegard-von-Bingen-Str. 1, Regensburg 93053, Germany

**Keywords:** composite scaffold, hyaluronic acid, gelatin, chondrogenesis, human mesenchymal stem cells

## Abstract

Mesenchymal stem cells (MSCs) seeded on specific carrier materials are a promising source for the repair of traumatic cartilage injuries. The best supportive carrier material has not yet been determined. As natural components of cartilage’s extracellular matrix, hyaluronic acid and collagen are the focus of biomaterial research. In order to optimize chondrogenic support, we investigated three different scaffold compositions of a hyaluronic acid (HA)-gelatin based biomaterial. Methods: Human MSCs (hMSCs) were seeded under vacuum on composite scaffolds of three different HA-gelatin ratios and cultured in chondrogenic medium for 21 days. Cell-scaffold constructs were assessed at different time points for cell viability, gene expression patterns, production of cartilage-specific extracellular matrix (ECM) and for (immuno-)histological appearance. The intrinsic transforming growth factor beta (TGF-beta) uptake of empty scaffolds was evaluated by determination of the TGF-beta concentrations in the medium over time. Results: No significant differences were found for cell seeding densities and cell viability. hMSCs seeded on scaffolds with higher ratios of HA showed better cartilage-like differentiation in all evaluated parameters. TGF-beta uptake did not differ between empty scaffolds. Conclusion: Higher ratios of HA support the chondrogenic differentiation of hMSCs seeded on a HA-gelatin composite scaffold.

## 1. Introduction

Treatment techniques for acute cartilage injuries range from debridement to microfracture to autologous cartilage transplantation. The choice of treatment is lesion-dependent, and it has been shown that larger lesions (>3.0 cm^2^) benefit from autologous cartilage implantation (ACI) especially in the long term [1,2], while smaller lesions can be treated by bone marrow stimulation techniques such as microfracture [3]. As the introduction of ACI [4] was a major step towards regenerative treatment of large chondral defects, it also revealed potential complications, such as hypertrophy of the periosteum, which is used to cover the injected autologous chondrocytes [5]. To overcome these problems, scaffolds have been developed to serve as a carrier for the autologous chondrocytes after *in vitro* expansion. The matrix-based ACI (MACI) could improve treatment and leave common complications behind [6,7]. However, there are still several disadvantages of the MACI therapy. The two most impactful of these are a two-step procedure that requires two operation time points and harvest of viable chondrocytes, thereby creating donor-site morbidity. Additionally, chondrocytes tend to dedifferentiate during monolayer culture prior to implantation [8]. In order to overcome these drawbacks, the call for alternative cell sources such as mesenchymal stem cells (MSCs) and smart biomaterials [9] is on the rise. Both have been under investigation by several working groups, and while chondrogenic differentiation of MSCs was established already in the late 90 s [10], clinical applications are still not well established. Recent studies show promising results of clinical application of MSCs for the cure of cartilage defects [11].

In order to further improve MSC-based repair of chondral defects, different materials have been employed for scaffold production. Hyaluronic acid (HA) is an important component of cartilage extracellular matrix, linking aggrecan molecules to large proteoglycans, and acts as a lubricant in diarthrodial joints. In clinical application, intra-articularly injected HA is used as a lubricant in degenerative osteoarthritis [12]. In tissue engineering, multiple working groups have shown that HA stimulates chondrogenesis of MSCs *in vitro* [13,14] mainly through interactions with cell receptors expressed by MSCs including CD 44 and CD168 [15,16]. Recent developments could generate native cartilage like properties of *in vitro* engineered constructs based on chondrogenic differentiation of MSCs in HA-based scaffolds [17]. As HA also promotes cell viability and migration, natural or synthetic biomaterials are combined with HA to improve the biocompatibility of the carrier [18]. Furthermore, HA can be modified in several ways. For example methacrylation allows for crosslinking by photopolymerization [19,20] or connection of additional extracellular, pro-chondrogenic signaling molecules such as N-cadherin [21]. This possibility functionalizes the HA-based scaffold towards the idea of a smart biomaterial. In order to achieve suitable primary biomechanical properties, HA needs to be crosslinked. However, chemically altered HA, e.g., crosslinked HA, first needs to prove its prochondrogenic capability as well as its advantages for cell migration.

The second major component of cartilage extracellular matrix (ECM) is collagen type 2, representing 95% of all collagens incorporated in cartilage ECM [22]. Therefore the addition of gelatin—a hydrolyzed derivative of collagen 1—to the scaffold matrix suggests more native-like properties for non-seeded scaffolds. However, gelatin is not capable of mimicking the function of native cartilage ECM as its structure and biological function differs from that of human collagen 2. Angele *et al.* [23] reported decreased biomechanical properties for tensile strength and Young’s modulus upon addition of gelatin to the composite scaffold. Additionally, cell adhesion was improved within scaffolds with higher ratios of gelatin. Gelatin is also metabolized to non-toxic degradation products.

The aim of the present study is to investigate the potential of composite scaffolds with three different HA/gelatin ratios to support chondrogenic differentiation of human MSCs (hMSCs) in order to improve the scaffold composition. Therefore we investigated the viability, gene expression patterns and production of cartilage-specific ECM of seeded hMSCs at different time points.

## 2. Results

### 2.1. Production of Hyaluronic Acid (HA)-Based Composite Scaffolds

**Figure 1:**
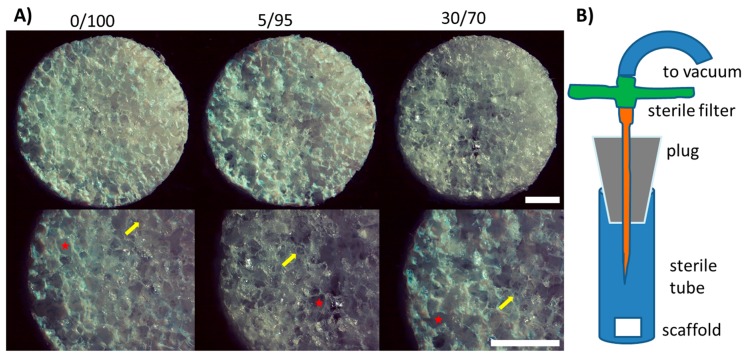
(**A**) Macroscopic imaging of scaffolds of different composition before seeding; primary and secondary pores show equal sizes. Stars indicate primary pores (250–300 µm in diameter), arrows point towards smaller secondary pores (50–100 µm); (scale bars = 1 mm); (**B**) Schematic of seeding of the scaffolds under vacuum conditions with hMSCs. The cell suspension is injected through the sterile filter before application of the vacuum.

**Figure A1:**
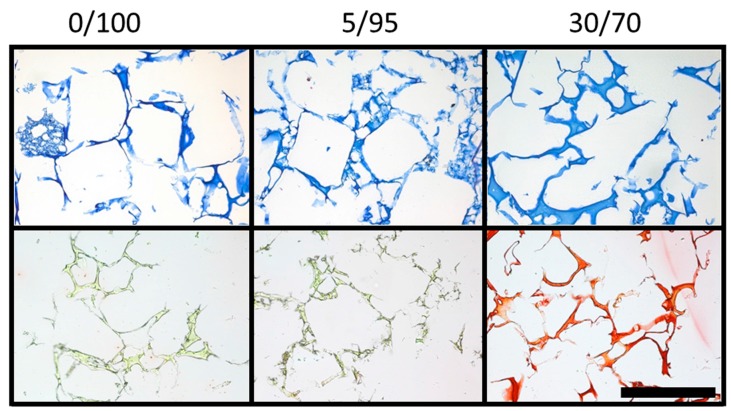
DMMB and picrosirius red staining of empty scaffolds of different composition (columns): DMMB staining for glycosaminoglycans (e.g., HA) at ×10 magnification (upper row) and picrosirius red staining for collagen (e.g., gelatin) content (scale bar = 500 µm). Scaffolds with higher gelatin content are more intensely stained by picrosirius red, while homogenous distribution of the components within the scaffolds is visualized.

In order to optimize chondrogenic differentiation of hMSCs for cartilage repair, we investigated three different compositions of a hyaluronic acid (HA)-based scaffold: 100% HA (0/100), 5% gelatin/95% HA (5/95) and 30% gelatin/70% HA (30/70). Scaffold production yielded macroscopically similar scaffolds with equal primary and secondary pore size between different scaffold compositions (Figure 1A) as reported previously [23]. Primary pores were approximately 250–300 µm in diameter, while secondary pores reached 50–100 µm in diameter (Figure A1). Scaffold dimensions were 5 mm in diameter and 2 mm in height, yielding a total volume of 50–60 µL.

### 2.2. Seeding, Adhesion and Viability of MSCs

**Figure 2:**
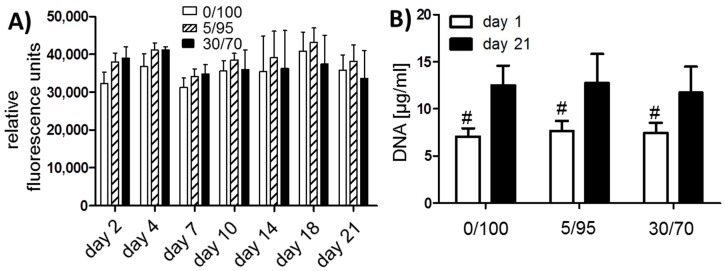

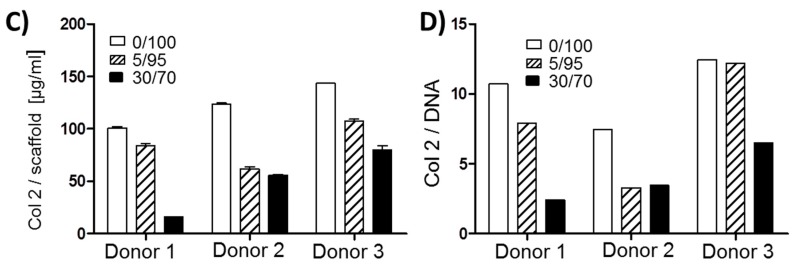
(**A**) Resazurin staining for viability of seeded MSCs; 5 scaffolds per donor and 3 donors per time point were evaluated; (**B**) DNA content of different scaffolds at day 1 and 21 shows no significant difference between scaffolds at the same time points (# *p* < 0.05 for all groups compared to day 21), 3 different donors per time point were evaluated; (**C**) Collagen 2 content per scaffold (µg/mL), primary hMSCs of three donors were analyzed at day 21; (**D**) Ratio of collagen 2 content per DNA, with higher ratios suggesting better chondrogenic differentiation. Primary hMSCs of three donors were analyzed at day 21.

Harvested hMSCs showed donor-dependent growth and were confluent within 10–12 days after plating. After one freeze-thaw cycle, MSCs again showed high viability and growth. At passage two scaffold seeding was successfully performed (Figure 1B) and viable MSCs could be detected within 48 h by resazurin staining (Figure 2A). Cell seeding density was approximately 20 × 10^6^ cells per mL. For long term assessment of cell viability, resazurin staining was performed on day 2, 4, 7, 10, 14, 18 and day 21. Cell viability did not differ significantly between groups and/or timepoints (Figure 2A). However 5/95 scaffolds showed the highest viability from day 10 on, while 30/70 scaffolds showed the highest viability in the beginning. Determination of DNA content revealed a significant increase from day 1 to day 21 within each scaffold composition. DNA content compared between scaffold compositions were not significantly different at day 1 and day 21 respectively, suggesting that the number of cells that adhere to or proliferate within the scaffold were not significantly different between groups (Figure 2B).

**Figure 3:**
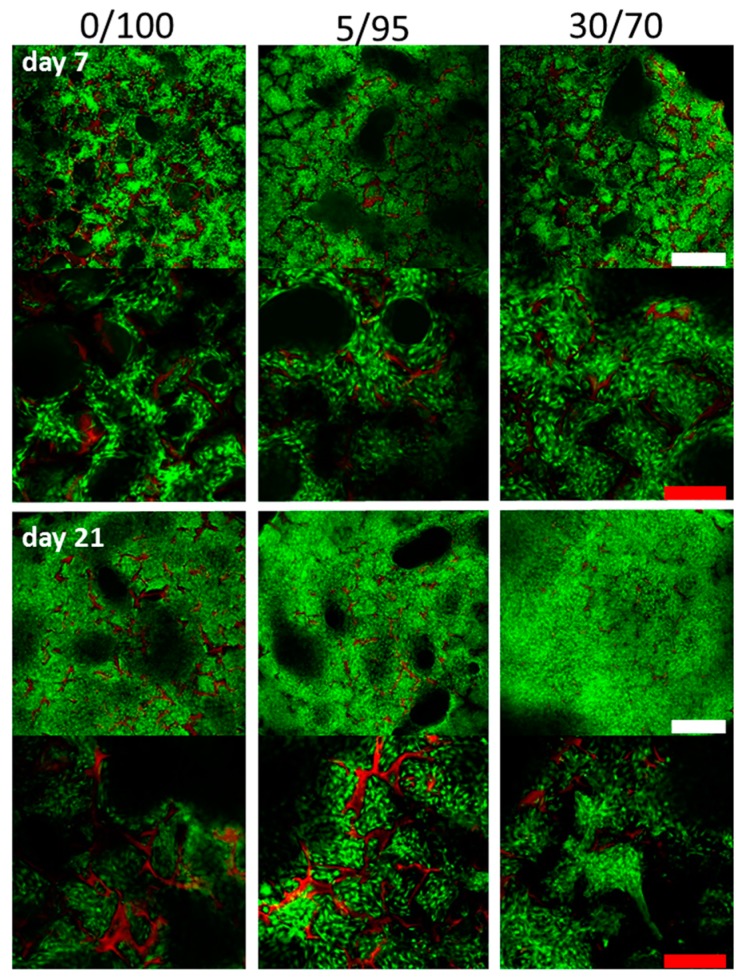
Confocal imaging after Calcein staining at day 7 and day 21 after cell seeding (upper rows ×4 magnification, lower rows ×10 magnification) depicting three dimensional adhesion of cells in primary and secondary pores in the center of the scaffolds. Visually, no differences between adhesion patterns and cell densities are detected. Green = Calcein-labeled cells; red = scaffold. (White scale bars = 500 µm, red scale bars = 250 µm).

Additionally confocal microscopy after Calcein staining for living cells was performed to allow for visualization of cell distribution within the scaffold (Figure 3). Again, no major differences were detectable between different scaffold compositions in terms of seeding density, cell distribution and viability. Interestingly, at day 7 some of the pores did not show any cell infiltration at all. This might be due to a complete occlusion of these pores. At day 21, these pores are not clearly demarcated when the scaffold is already partially degrading, allowing cells to infiltrate the initially occluded pores.

### 2.3. Chondrogenic Differentiation of Seeded MSC—Gene Expression Analysis and Extracellualar Matrix Production

The main aim of this study was to determine which scaffold material will best support chondrogenic differentiation of hMSCs. To answer this question, all three different scaffold compositions were seeded with the same hMSCs (three different donors, not pooled) and evaluated for chondrogenic differentiation and production of cartilage specific ECM.

In order to evaluate for ECM production, scaffolds were harvested after 21 days of culture in chondrogenic medium and analyzed for DNA and collagen 2 content. No significant differences were observed for DNA content (Figure 2B). For all three primary hMSC cell lines differences could be detected for collagen 2 content per matrix (Figure 2C) as well as for collagen 2 per DNA (Figure 2D). In all three hMSC cell lines 0/100 scaffolds yielded best collagen 2 production. hMSCs in 5/95 scaffolds also showed higher collagen 2 production than in 30/70 scaffolds with exemption for donor 2, while 30/70 scaffolds showed the lowest cartilage-specific ECM production.

**Figure 4:**
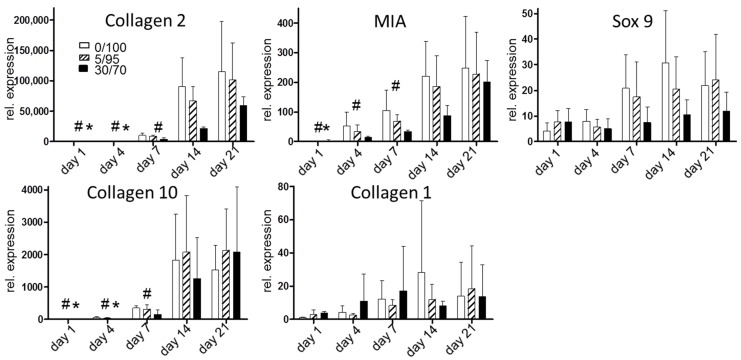
Gene expression analysis during chondrogenic differentiation for chondrogenic differentiation markers (upper row) and markers of hypertrophy (Collagen 10) and fibrous differentiation (Collagen 1) (# *p* < 0.05 for all groups compared to day 21, * *p* < 0.05 for all groups compared to day 14). Rel. gene expression = compared to mean gene expression of housekeeping genes.

In order to evaluate for chondrogenic differentiation, expression of cartilage-specific genes was evaluated (Figure 4). Again, collagen 2 expression levels were highest in 0/100 scaffolds followed by 5/95 scaffolds, while 30/70 scaffolds showed lower expression levels at all time points. No statistical significant differences were observed between scaffolds within one time point. Melanoma inhibitory activity protein (MIA) as a marker of chondrogenic differentiation [24] showed higher levels in 0/100 scaffolds compared to 5/95 and 30/70 scaffolds. However, at day 1, MIA reached its highest levels in 30/70 scaffolds and differences between the scaffolds were not as clear as for collagen 2 expression levels. We further analyzed Sox 9 gene expression levels as a third marker of chondrogenic differentiation and found similar results as for collagen 2 and MIA. Again, expression levels of the chondrogenic marker Sox 9 were not as clearly different between higher proportions of HA scaffolds as for collagen 2 expression, but were still higher than for hMSCs seeded on 30/70 scaffold, and again at day 1 Sox 9 was highest in 30/70 scaffolds (Figure 4).

In order to crosscheck for hypertrophy in chondrogenic differentiated MSCs, we evaluated the gene expression of collagen 10, a well known marker of chondrocyte hypertrophy [25]. Here we found similar expression levels for all three scaffolds up to day 4, while 0/100 and 5/95 scaffolds showed more hypertrophic expression at day 7 and 14. 5/95 and 30/70 scaffolds showed the highest expression levels of collagen 10 at day 21. To characterize gene expression of fibrous markers we evaluated collagen 1 expression levels. Here we could detect more fibrous differentiation of hMSCs in 30/70 scaffolds at day 1 through 7 while at day 14 collagen 1 expression was highest in 0/100 scaffolds and at day 21 in 5/95 scaffolds. No statistically significant differences were found.

Taking the results of the gene expression analysis and evaluation of cartilage specific ECM production together, hMSCs are more likely to differentiate towards a chondrogenic geno- and phenotype when seeded on scaffolds with higher proportions of HA.

### 2.4. Histomorphometric Appearance

**Figure 5:**
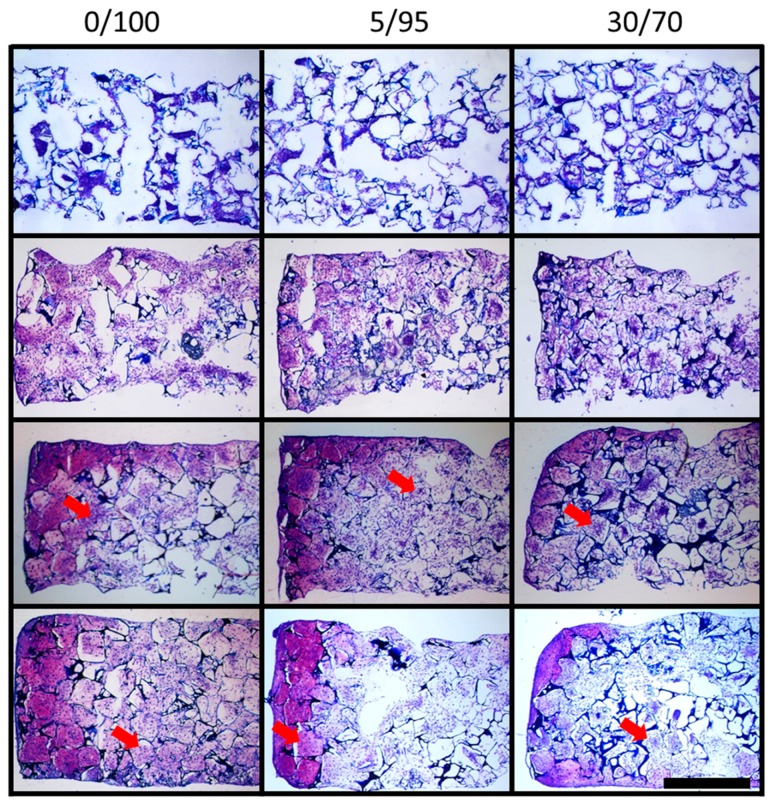
Dimethylmethylene blue (DMMB) staining for glycosaminoglycan content of MSC seeded scaffolds of different ratios of hyaluronic acid and gelatin throughout chondrogenic differentiation: first row day 1, second row day 7, third row day 14 and fourth row day 21 (scale bar = 1 mm). Arrows indicate ECM in the lumen of the scaffold pores, located pericellular to intraluminal MSCs.

At days 1, 7, 14 and 21 scaffolds were stained by dimethylmethylene blue (DMMB) to detect glycosaminoglycans (GAG), a major composite of cartilage-specific ECM. Scaffolds showed primary and secondary pores due to the two-step scaffold production using porogenic grains and air insufflation (Figure 5, top row). At day 1 some pores seem to be filled by cells that line up in chains, while others show hardly any cells. Day 7, 14 and 21 samples show equal cell distribution throughout the lumen of all primary and secondary pores (see Figure 5). Cell-free pores at day 1 might be due to missing cell attachment, missing ECM and wash-out of cells during slide preparation.

As expected, GAG content increased over time within every scaffold composition. While on day 1, strong blue staining of the scaffold material is visible due to the high content of HA as source material of the scaffolds, overall staining further increased over time and is found to be more and more pericellular, and in the lumen of the scaffolds’ pores rather than at the wall of the pores as ECM is produced by invaded MSCs (Figure 5). It is notable that 0/100 and 5/95 scaffolds show more intense staining at day 14 and 21 than 30/70 scaffolds, suggesting poorer chondrogenic differentiation of hMSCs in 30/70 scaffolds (Figure 5).

### 2.5. Immunohistochemistry

**Figure 6:**
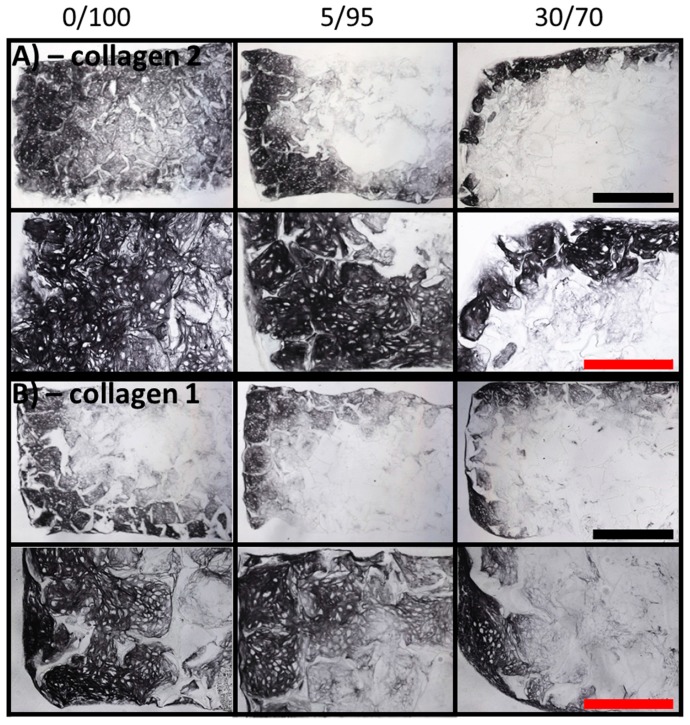
Immunohistochemistry of MSC-seeded scaffolds of different composition (columns) after 21 days of culture in chondrogenic medium. (**A**) Immunohistochemistry for collagen 2 at ×4 magnification (upper row, black scale bar = 1 mm) and ×10 magnification reveals more collagen 2 deposition in 0/100 scaffolds than in 30/70 scaffolds (red scale bar = 500 µm). Best samples are shown; (**B**) Immunohistochemistry for collagen 1 at ×4 magnification (upper row, black scale bar = 1 mm) and ×10 magnification reveals no major differences of collagen 1 deposition between the three tested scaffold compositions; best samples are shown (red scale bar = 500 µm).

To evaluate differences in collagen 2 amount—the main collagen type in cartilage specific ECM—samples were subjected to immunohistochemistry at day 21. Again, primary and secondary pores were filled by ECM-producing chondrogenic differentiated MSCs, but homogenous deposition of collagen 2 was only to be found in 0/100 and 5/95 scaffolds (Figure 6A). Scaffolds consisting of 30% gelatin and 70% HA only showed staining for collagen 2 at the periphery of the scaffold (Figure 6A, right hand column). At higher magnifications, chondrocyte-like phenotypes could be seen in all collagen 2 positive areas.

Additionally, immunohistochemistry for collagen 1 was performed at day 21 to detect possible de-differentiation of seeded MSCs towards a more fibrous phenotype. Collagen 1 could be detected in all scaffold compositions at peripheral areas, while the centers of the scaffolds did not show major collagen 1 deposition (Figure 6B). Again, chondrocyte-like phenotypes were detected in all collagen 1 positive areas at higher magnifications, suggesting enhanced Col 1 production in areas of increased chondrogenic differentiation.

### 2.6. TGF-Beta Concentrations in Supernatant under Cell Free Scaffold Conditions

**Figure 7:**
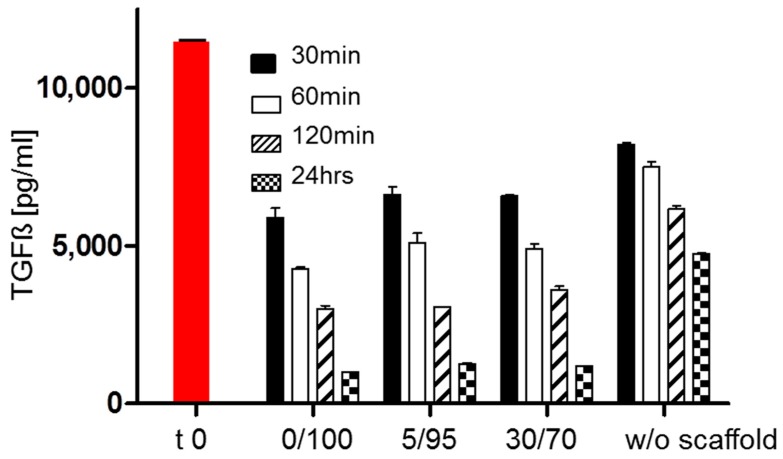
TGF-beta concentrations in medium containing empty scaffolds of different compositions and without any scaffold (w/o scaffold); red bar time zero; no significant differences were seen between scaffolds at same time points.

As one possible reason for differences in chondrogenic differentiation is different availability of TGF-beta, the most potent contributor to chondrogenic differentiation *in vitro*. We tested whether different scaffold compositions would alter the availability of TGF-beta. The performed enzyme-linked immunosorbent assay (ELISA) for TGF-beta 1 did not show significant differences between scaffolds (Figure 7), while concentrations in the scaffold free control were higher. This firstly suggests no differences of TGF-beta binding or inactivation by different scaffold compositions and secondly shows that already cell free scaffolds lower the amount of free TGF-beta compared to medium only. The time-dependent loss of active TGF-beta is already well known with different half-life times of the protein reported *in vitro* and *in vivo*.

## 3. Discussion

A HA-gelatin composite scaffold was used in this study to chondrogenically differentiate attached hMSCs and thus grow cartilage tissue constructs *in vitro*. It was shown that not only can cartilage and osteochondral repair tissue be grown on this composite matrix [26], but also recently fibrocartilaginous meniscus repair tissue was generated for *in vivo* applications [27,28]. Characterization of matrix properties showed that addition of gelatin to HA-based scaffolds would enhance cell adhesion but decrease mechanical properties [23], while scaffolds containing larger pores showed better mechanical properties in terms of maximum stress at rupture and Young’s modulus [29]. Most recently, Matsiko *et al.* [30] showed that scaffolds containing pore sizes up to 300 µm support chondrogenic differentiation better than scaffolds containing smaller pore sizes. According to this finding, our scaffold with an average primary pore size of 250–300 µm diameter will stimulate chondrogenic differentiation by means of the scaffold microarchitecture. However, it was not clear which ratio of the scaffold components will reinforce chondrogenic differentiation of hMSCs best. The three different scaffold compositions investigated to that end in the current study showed better chondrogenic differentiation in all evaluated outcomes for higher ratios of HA, e.g., 100% HA compared to 30% gelatin/70% HA scaffolds. Similar results were obtained for all three scaffold compositions for appearance under confocal microscopy, cell viability and DNA content at early time points, suggesting that there were no major differences in primary cell adhesion between gelatin containing scaffolds—30/70 or 5/95—and HA only scaffolds. The estimated overall seeding density of hMSCs reached 20 × 10^6^ cells/mL within each scaffold composition. Studies investigating the optimized seeding density in HA hydrogels found that hydrogels seeded with 60 × 10^6^ cells/mL reached close to native cartilage biomechanical and biochemical properties [17]. Therefore, we think that increase of cell seeding density might result in more homogenous ECM production. However, the same study showed significant differences only for mid-term culture duration (e.g., 56 days) while short term culture (e.g., 28 days) revealed no significant difference between 60 × 10^6^ cells/mL and 20 × 10^6^ cells/mL.

As revealed by confocal microscopy (Figure 3) some pores were not infiltrated at all by cells at day 7 in all three investigated scaffold compositions. We think that in these cases the pores were perfectly closed off from the surrounding environment and therefore did not allow for MSC infiltration. This finding was revised towards longer culture time with pore walls being gradually degraded allowing cells to migrate into the empty space and therefore building more homogenous ECM and more homogenous cell distribution. This finding was also observed in histology of DMMB stained slides, where the time dependency of the improvement of ECM distribution is even better visualized (Figure 5). DMMB staining also showed higher proportions of GAG (sulfated and non-sulfated), providing evidence for increased, cartilage-specific ECM production. Interestingly, staining intensity decreases for 5/95 and 30/70 scaffolds from day 14 to day 21. One possible explanation for this phenomenon is beginning hypertrophic conversion of chondrogenically differentiated MSCs with concomitant ECM degradation. However, DMMB stains have to be interpreted carefully as HA incorporated in the scaffold will also be stained by DMMB.

Former studies reported on *in vitro* degradation times of derivatized hyaluronic ester of 2 months [31] and more rapid degradation of gelatin [32]. Both studies did not investigate degradation of HA and gelatin in the used combination, so that a concrete degradation profile of both components within the scaffold needs to be investigated in further studies. The substitution of scaffold material by extracellular matrix as evidenced in our study is in accordance to previous reports and beneficial for restoration of native tissue.

Besides these favorable features, cell-produced ECM was mainly detected at the edge of the scaffold. A possible reason for this might be critical shortage of nutrients in the center of the scaffolds. A recent study found decreased oxygen supply as a main cause and was able to improve nutrient supply by higher perfusion rates in tantalum-based scaffolds for bone tissue engineering [33]. We think that an additional solution will be improved 3-D architecture of the scaffolds. As one of our previous observations using SEM [23] as well as our confocal images suggest that some of the pores are entirely closed preventing cell invasion. One solution to open up all pores might be to use different grain sizes to produce some larger primary pores or to introduce drainage channels to enable access for cells and nutrients to the scaffold’s center. These approaches have to be tested in future investigations.

Furthermore, it was shown nearly three decades ago that HA stimulates chondrogenic differentiation of MSCs [13]. Therefore, HA was used in multiple tissue engineering approaches ranging from HA based polymers (e.g., Hyaff 11) [34] to hydrogel [20,35] to fibrous scaffolds [36]. Whether used *in vitro* or *in vivo*, HA based cartilage tissue engineering was capable of restoring cartilage-like tissue. The prochondrogenic effect of HA [14] is mainly mediated by cell receptors expressed by MSCs including CD 44 and CD168 [15,16]. Therefore the superiority of higher HA ratios for chondrogenic differentiation in the investigated composite scaffolds is not too surprising, but nevertheless other effects must not be neglected. For instance absorption or inactivation of TGF-beta—one of the main pro-chondrogenic stimulators [10]—might be different within different scaffold compositions. Testing for the amount of available TGF-beta in culture medium revealed no differences between different scaffolds, but was definitely showing TGF-beta loss over time. This is contributed to the short half life time of TGF-beta, but also contributed to TGF-beta absorption of the polystyrene dish as similar absorption/inactivation kinetics were observed for scaffold-free medium (Figure 7).

In clinical application, different microarchitectural scaffold designs and scaffold compositions are used ranging from collagen-based fleeces to collagen-based sponges or HA based webs. In a recent descriptive study of four clinically used scaffolds, it was shown that cell distribution is impacted by fiber density (especially inter-fiber space smaller 100 µm impact cell distribution), cell phenotype is determined by fiber size and cell adhesion is mainly influenced by material composition and its structure [37].

Additional investigations need to be performed in order to fully characterize biophysical and biochemical properties of the proposed cartilage constructs, optimize culture conditions, e.g., mechanical stimulation, and evaluate optimal time of implantation for further translational *in vivo* evaluation.

## 4. Materials and Methods

### 4.1. Isolation and Culture of MSCs

Bone marrow derived hMSCs were obtained from patients undergoing surgery with bone harvest from the iliac crest after preoperative informed consent and approval by the local ethical committee. MSCs were aspirated with a heparinized syringe. After addition of Dulbecco’s modified Eagle’s medium (DMEM), low glucose concentration (5%), with 10% fetal bovine serum, 1% penicillin, and 1% Hepes buffer nucleated cells were plated in culture dishes at a density of 2 × 10^6^ cells/75 cm^2^ styrene coated culture dish. After adhesion, medium was changed twice a week and cells were trypsinized and deep frozen after 80% confluence was reached.

### 4.2. Production of Scaffolds

For producing the porous scaffolds we used solvent casting and particulated leaching as described previously [23]: As source material we used derivatized hyaluronic acid commercially available as wound dressing film Jaloskin (Fidia Advanced Biopolymers, Abano Terme, Italy). Hereby hyaluronic acid (HA) is highly esterified by benzylalcohol on its free carboxyl groups of the glucuronic acid along the backbone of the polymer. The gelatin component of the scaffolds was produced of hydrolyzed porcine collagen type 1 (Sigma-Aldrich, Taufkirchen, Germany). The components have been dissolved in hexafluoroisopropanol (HFIP, Sigma-Aldrich, Taufkirchen, Germany). To create primary pores we used Sodium Chloride at a grain size of 250–300 µm. Secondary pores of 50–100 µm in size have been generated in the biomaterial by air insufflation between the porogenic grains during the evaporation process of the solvent. For our study purposes we produced scaffolds consisting of 100% HA (0/100), 5% gelatin and 95% HA (5/95) and scaffolds consisting of 30% gelatin and 70% HA (30/70). Finished scaffolds were on average 5 mm in diameter and 2 mm in height (Figure A1).

### 4.3. Seeding and Culture of MSCs in Composite Scaffolds

Produced scaffolds were sterilized by beta-irradiation and after thawing, hMSCs were re-seeded, expanded and harvested again at 80% confluence. Sterile scaffolds were cell-seeded by adding 1 × 10^6^ MSCs by injecting onto the cylindrical polymer scaffolds. Seeding density reached approximately 20 × 10^6^/mL as the volume of one scaffold contains 50–60 µL. Seeding was performed under vacuum conditions (−1 bar, 10 s) to guarantee good infiltration and distribution of the cells into the porous scaffold as previously described [38]. After that, scaffolds were transferred to a 24 well plate and cell adhesion was allowed for one hour. Cell-seeded scaffolds were cultured at 37 °C in 5% CO_2_ in Dulbecco’s modified Eagle’s medium (DMEM), high glucose with, 1%ITS+3, Dexamethasone (0.1 µM), ascorbic acid (200 µM), pyruvate (0.11 mg/mL). Each group was cultured with addition of 10 ng/mL TGF-beta 1 (R & D Systems, Wiesbaden, Germany) to induce chondrogenesis. Medium was changed 3 times per week.

### 4.4. Cell Viability Measurement

To answer the question whether different scaffold compositions influence cell viability in a different way we used a resazurin based viability assay (Sigma-Aldrich, Taufkirchen, Germany). After permeating into the cell the resazurin-dye undergoes an oxidation-reduction reaction as a result of an intracellular metabolic reaction therefore indicating living cells. In short, cell seeded scaffolds were incubated (45 min; medium/resazurin = 10/1) according to the manufacturer’s instructions and relative fluorescence units of the supernatant were determined using excitation wavelengths of 545 nm and detection wavelengths of 590 nm. Cell viability was assessed at day 2, 4, 7, 10, 14, 18 and day 21. Cell viability is not affected by this assay [39].

### 4.5. Gene Expression Analysis

For gene expression analysis, samples were homogenized in RNEasy Plus Universal (Quiagen, Hilden, Germany) reagent with a tissue grinder, RNA was extracted according to the manufacturer’s instructions, and the RNA concentration was determined using a spectrophotometer (Nanodrop ND-2000, Fisher Scientific, Schwerte, Germany). One microgram of RNA from each sample was reverse transcribed into cDNA using Transcriptor First Strand Synthesis Kit (Roche, Mannheim, Germany) and cDNA amplification was performed using a Biorad CFX 96 real-time polymerase chain reaction system with intron-spanning primers and SYBR Green Reaction Mix (Agilent, Santa Clara, CA, USA). The relative gene expression was calculated using the delta-delta-Ct algorithm (DDCT method). Each sample was normalized to the average expression of the three housekeeping genes: Receptor expression-enhancing protein 5 (REEP-5), vacuolar protein sorting protein (VPS-29), Proteasome subunit beta type-4 (PSMMB-4). Expression levels of the cartilage-specific markers Sox 9, Collagen II, and MIA, and the hypertrophy-related marker, Collagen-type X and the fibrous-tissue related marker Collagen I, were determined and relative gene expression compared to mean gene expression of housekeeping genes was reported. For complete sequences of all primers see Table A1.

### 4.6. Histology

For histomorphometry cell-seeded constructs were harvested on days 1, 7, 14 and 21 and frozen sections were prepared. Samples were cut to 10 µm thin sections and stained with dimethylmethylene blue (DMMB) to detect glycosaminoglycans and microscopically evaluated.

### 4.7. Immunohistochemistry

Sectioned samples (10 µm thickness) were rehydrated in washing buffer (TRIS 0.2 M, NaCl) for 10 min and peroxidases blocked by PBS containing 10% H_2_O_2_ and 10% Methanol for 30 min. For antigen retrieval samples were incubated by 0.1% pepsin at pH 3.5. After applying monoclonal primary antibodies against collagen I (Sigma-Aldrich, Taufkirchen, Germany) and II (Merck, Darmstadt, Germany), biotin conjugated polyclonal secondary antibodies (goat anti-mouse IgG (Jackson, West Grove, PA, USA)) and the nickel and cobalt enhanced DAB stain were used for visualization. Samples were microscoped and photographed using a Nikon Eclipse microscope (Nikon, Düsseldorf, Germany).

### 4.8. Confocal Microscopy

For visualization of cell distribution within the 3D space of the scaffold, we used confocal microscopy after fluorescence labelling of the viable cells. Therefore we used standard protocols for Calcein AM labelling according to the manufacturer (LIVE/DEAD Viability/Cytotoxicity Kit, Mo Bi Tec, Göttingen, Germany). Scaffolds were examined under the confocal microscope (Nikon D Eclipse C1, Nikon, Düsseldorf, Germany) and photographed at 4× and 10× magnification.

### 4.9. Analysis of Extracellular Matrix Production

To assess capability of extracellular matrix production an enzyme-linked immunosorbent assay (ELISA) test for collagen II was performed. MSC-seeded scaffolds were homogenized (0.05 M acetic acid plus 0.5 M NaCl (pH 2.9–3.0)), digested with 10 mg/mL pepsin and dissolved in 0.05 M acetic acid under rotation for 48 h at 4 °C, followed by elastase digestion for 24 h. According to the manufacturer’s protocol (Native Type II Collagen Detection Kit 6009, Chondrex, Redmond, WA, USA) digestion and the collagen type II estimation were performed. DNA concentrations were assayed using the Quant-iT PicoGreen dsDNA Assay Kit (Invitrogen, Eugene, OR, USA). Collagen-type II and DNA content were determined and chondrogenic capability was defined as the ratio between content of collagen type II and DNA for each MSC-seeded scaffold.

### 4.10. Analysis of TGF-Beta Concentrations in Supernatant in Cell Free Scaffold Conditions

As one possible reason for different chondrogenic potential of hMSCs within different scaffold formulation might be differences in availability of active TGF-beta as the most prochondrogenic stimulator. We tested whether different scaffold compositions might alter the availability of TGF-beta. Therefore empty scaffolds of the three different compositions (0/100, 5/95 and 30/70) were incubated in prochondrogenic medium as described under Section 4.3. At 0, 30, 60 and 120 min as well as after 24 h medium was collected. According to the manufacturer’s protocol (TGF ß1–ELISA, Duoset, R & D Systems, Minneapolis, MN, USA) the TGF-beta concentration was determined by quantitative ELISA.

### 4.11. Staistical Analysis

Statistical analysis was carried out using SPSS^®^ (version 20, IBM, Armonk, NY, USA). We applied the Kolmogorov-Smirnov test to check for normal distributions. For normally distributed items a two-way analysis of variance (ANOVA) with group and time as independent factors followed by individually performed posthoc tests to maintain overall alpha level at *p* < 0.05 was performed. For non-normal distributions, a Kruskal-Wallis test followed by individual Mann-Whitney test was used. To control for type-1 error, a Bonferroni correction for multiple comparisons was applied.

## 5. Conclusions

A HA-gelatin composite scaffold is suitable for cartilage tissue engineering. Three different scaffold compositions with different ratios of HA/gelatin showed different promotion of chondrogenic differentiation of hMSCs. In our study, scaffolds with 100% HA were more supportive for chondrogenic differentiation than scaffolds with lower ratios of HA. In future work, improvement of 3D scaffold design and functionalization of HA will be investigated.

## Abbreviations

The following abbreviations are used in this manuscript:

MSC	mesenchymal stem cell
ELISA	enzyme-linked immunosorbent assay
ANOVA	analysis of variance
RNA	ribonucleic acid
DNA	desoxy-ribonucleic acid
HA	hyaluronic acid
TGF-beta	transforming growth factor beta

## Appendix

**Table A1:** PCR-Primers and sequences.

Primer	Forward	Reversed
Collagen 2	5′ GGGCAATAGCAGGTTCACGTA 3′	5′ TGTTTCGTGCAGCCATCCT 3′
Collagen 1	5′ ACGTCCTGGTGAAGTTGGTC 3′	5′ ACCAGGGAAGCCTCTCTCTC 3′
Collagen 10	5′ CCCTCTTGTTAGTGCCAACC 3′	5′ AGATTCCAGTCCTTGGGTCA 3′
Sox 9	5′ ACACACAGCTCACTCGACCTTG 3′	5′ AGGGAATTCTGGTTGGTCCTCT 3′
MIA	5′ AAAGGGGTCATCGTAACAGG 3′	5′ GGGAAGTCGAACCTCTTCTG 3′
PSMB 2	5′ GCTGCCAGGTAGTCCATGTAA 3′	5′ CGAAACCTGGCTGACTGTCT 3′
REEP 5	5′ AGGTCAGCCACTGGGTATCA 3′	5′ CCTCTCTCCTCTGCAACCTG 3′
VPS 29	5′ AGCTGGCAAACTGTTGCAC 3′	5′ GACGGTGGTGGTGACTGAG 3′
